# Outpatient prescribing pattern for acute bronchitis in primary healthcare settings in China

**DOI:** 10.1038/s41533-021-00234-y

**Published:** 2021-05-10

**Authors:** Mengyuan Fu, Haishaerjiang Wushouer, Lin Hu, Nan Li, Xiaodong Guan, Luwen Shi, Dennis Ross-Degnan

**Affiliations:** 1 Department of Pharmacy Administration and Clinical Pharmacy, School of Pharmaceutical Sciences, Peking University, Beijing, China; 2 Department of Population Medicine, Harvard Medical School and Harvard Pilgrim Health Care Institute, Boston, MA USA; 3 International Research Center for Medicinal Administration, Peking University, Beijing, China; 4 Clinical Epidemiology Research Center, Peking University Third Hospital, Beijing, China

**Keywords:** Drug regulation, Health services

## Abstract

Inappropriate prescribing for acute bronchitis in primary healthcare settings (PHSs) is commonly seen worldwide. Here we describe the prescribing patterns and antibiotic use for acute bronchitis in PHSs across China. We conduct a nationwide cross-sectional survey to collect outpatient prescriptions from PHSs in 2017. Patients diagnosed with acute bronchitis without other infections are eligible for this study. Generalized estimating equations are used for analysis. Overall, 10,678 prescriptions for acute bronchitis from 214 institutions are included. The antibiotic prescription rate is 44.5% for total prescriptions, and differs significantly by region and urban/rural status (*p* < 0.05). Among all single-antibiotic prescriptions, 91.5% are broad-spectrum. Two-thirds of the prescriptions contain medicines for symptom management. The overall guideline compliance rate of acute bronchitis treatment for adults is 31.0%. Prescribing antibiotics, especially broad-spectrum ones, for acute bronchitis is commonly observed in Chinese PHSs. Targeted interventions are urgently needed for Chinese primary clinicians, especially in western rural areas.

## Introduction

Acute bronchitis, an inflammation of the lower respiratory tract manifested by acute cough, is among the commonest clinical condition responsible for primary healthcare consultations^1,2^. It is estimated that every year, 5% of the general population report an episode of acute bronchitis, 90% of whom seek medical advice^3^. Acute bronchitis is a self-limited condition caused by virus in at least 90% of cases, in which antibiotics have no role in treatment^4,5^. However, studies show that most patients with acute bronchitis are treated with inappropriate or ineffective therapies^6^. Antibiotics are inappropriately prescribed for acute bronchitis up to 70% of the time, accounting for 44% of all outpatient antibiotic prescriptions in the US^4^. Unnecessary antibiotic prescriptions provide no clinical improvement^3^, increase the prevalence of antibiotic resistance^7^, and healthcare costs^8^. Symptoms of acute bronchitis, such as acute cough and sputum production, generally last for 2–3 weeks^1,3,5^. Clinicians are challenged with providing evidence-based and effective symptom control therapies as the viral syndromes progress.

Reducing overprescribing of antibiotics for respiratory infections has been emphasized in China in the past decade to tackle antibiotic resistance^9–12^. Previous study showed that 93.5% of acute bronchitis visits in Chinese primary healthcare settings involved an antibiotic prescription, among which more than 50% were inappropriate^9^. However, studies are limited on the rates of antibiotic use and antibiotic selection for acute bronchitis in primary healthcare settings in China, as well as the detailed symptom treatment strategies for acute bronchitis. Therefore, we designed this study to evaluate outpatient antibiotic utilization, predictors of antibiotic use, and overall prescribing patterns for acute bronchitis at primary healthcare in China.

## Results

### Description of demographic characteristics

A total of 10,678 prescriptions for acute bronchitis from 214 Chinese primary healthcare settings in 37 cities in eight provinces were eligible for inclusion in our study (Table 1). After weight adjustment, 57.3% of the prescriptions were from urban community healthcare centers and 42.5% were from rural township hospitals; 24.9%, 22.8%, 22.9%, and 29.4% of the prescriptions were from the cities that in lowest to highest levels of economic status, respectively. Most prescriptions were from the eastern region (57.4%), followed by central (25.4%) and western region (17.2%); 52.7% of the patients were females and 30.7% were aged 18–64 years.

**Table 1:** Characteristics and antibiotic use for acute bronchitis at primary healthcare facilities in China.

Characteristics	Original sample size		Weighted sample size		Generalized estimating equations			APR
	Prescriptions	%	Prescriptions	%	Coefficients	95% CI	*p*	(Adjusted)
**Area**								
Urban	5369	50.3	6134.0	57.5	Reference			42.3
Rural	5309	49.7	4542.3	42.5	1.112	(1.005–1.219)	0.039	53.6
**Economic status**								
Low	1573	14.7	2659.5	24.9	Reference			50.3
Middle low	4414	41.3	2435.4	22.8	0.986	(0.859–1.114)	0.835	49.0
Middle High	762	7.1	2447.8	22.9	0.990	(0.852–1.128)	0.890	49.4
High	3929	36.8	3133.6	29.4	0.908	(0.763–1.054)	0.216	41.1
**Region**								
Eastern	6111	57.2	6132.3	57.4	Reference			41.3
Central	1435	13.4	2710.4	25.4	1.148	(1.025–1.271)	0.019	56.0
Western	3132	29.3	1833.5	17.2	1.122	(0.928–1.315)	0.218	53.4
**Patient gender**								
Female	5597	52.4	5630.3	52.7	Reference			47.6
Male	5081	47.6	5045.9	47.3	0.990	(0.953–1.027)	0.601	46.6
**Patient age**								
0–5	810	7.6	746.6	7.0	Reference			44.3
6–17	487	4.6	464.2	4.3	1.058	(0.981–1.135)	0.139	50.1
18–64	3211	30.1	3274.0	30.7	1.046	(0.973–1.119)	0.216	48.9
65–79	1467	13.7	1447.7	13.6	1.006	(0.920–1.093)	0.890	44.9
80+	562	5.3	532.4	5.0	0.951	(0.876–1.027)	0.205	39.4
Unknown	4141	38.8	4211.4	39.4	1.034	(0.918–1.150)	0.564	47.7
Total	10,678	100.0	10,676.3	100.0	–	–	–	–

### Antibiotic prescribing and predictors

After weighting to better reflect the national income and urban/rural distribution, the antibiotic prescription rate (APR) for acute bronchitis was 44.5% (unadjusted for other variables). After further adjustment (Table 1), clinicians in community centers prescribed antibiotics for acute bronchitis less frequently than those in township hospitals (42.3% vs. 53.6%, coefficients = 1.112, *p* = 0.039). The facilities in cities of the highest economic status had lower adjusted APR (41.1%) than those in the lowest economic status (50.3%) although the difference was not statistically significant (coefficients = 0.908, *p* = 0.216). Facilities located in eastern region (41.3%) had significantly lower adjusted APR than those in central region (56.0%, coefficients = 1.148, *p* = 0.019) and non-significantly lower APR than western region (53.4%, coefficients = 1.122, *p* = 0.218). Economic status, patient gender, and patient age were not the significant predictors of antibiotic prescribing.

### Distribution of prescribed antibiotic by drug classes

Of all the antibiotic prescriptions for acute bronchitis, 12.0% contained more than one antibiotic (Table 2). Township hospitals had significantly higher multi-APR than community centers (15.7% vs. 7.7%, *p* = 0.000). Among all single-antibiotic prescriptions, 91.5% were broad-spectrum. Township hospitals had significantly higher rate of broad-spectrum antibiotics than community centers (96.8% vs. 86.5%, *p* = 0.000). The most commonly prescribed antibiotic classes in urban areas were second-generation cephalosporins (31.8%), macrolides (19.8%), and third-generation cephalosporins (19.6%). In contrast, third-generation cephalosporins (23.1%) were identified as the most commonly used antibiotic in rural areas, followed by macrolides (19.4%) and broad-spectrum penicillins (16.9%). In both urban and rural areas, the most commonly used second-generation cephalosporins were cefuroxime (66.2% vs. 46.1%, respectively) and cefaclor (24.8% vs. 43.0%, respectively). The most commonly used third-generation cephalosporins were cefixime, cefdinir, and ceftazidime in urban areas (31.7%, 24.7%, 23.3%), and ceftazidime, cefotaxime, and ceftriaxone in rural areas (46.9%, 22.8%, 15.7%).

**Table 2:** Antibiotics prescribed for acute bronchitis at primary healthcare facilities in China, by urban–rural location and antibiotic type.

Antibiotic subgroup/substance	Prescription rate (weighted, %)^a^			
	Total	Urban	Rural	*p*
**Single-antibiotic prescriptions**	88.0	92.3	84.3	***
Narrow spectrum	8.5	3.2	13.5	***
First-generation cephalosporins	4.9	1.1	8.7	***
Narrow-spectrum penicillins	3.1	2.0	4.1	***
Nitroimidazoles	0.4	0.2	0.7	***
Broad-spectrum	91.5	96.8	86.5	***
Second-generation cephalosporins	22.4	31.8	13.3	*
Third-generation cephalosporins	21.4	19.6	23.1	***
Macrolides	19.6	19.8	19.4	***
Broad-spectrum penicillins	14.7	12.4	16.9	***
Quinolones	7.2	7.5	7.0	***
Lincosamides	5.0	5.3	4.6	***
Aminoglycosides	0.7	0.0	1.4	***
Other	0.6	0.4	0.8	***
**Multi-antibiotic prescriptions**	12.0	7.7	15.7	***
Third-generation cephalosporins+quinolones	20.0	10.7	24.0	***
Second-generation cephalosporins+quinolones	11.5	18.2	8.5	***
Broad-spectrum penicillins+quinolones	8.5	3.5	10.7	**
Macrolides+quinolones	7.2	18.9	2.1	***
Macrolides+lincosamides	6.2	18.2	0.9	***
Third-generation cephalosporins+broad-spectrum penicillins	4.6	0.0	6.7	***
Second-generation cephalosporins duplications	4.4	6.2	3.6	
Second-generation+third-generation cephalosporins	4.3	0.0	6.2	***
Other	33.3	24.2	37.3	**
Total	100.0	100.0	100.0	

*p < 0.05; **p < 0.01; ***p < 0.001.

^a^For single-antibiotic prescriptions or multi-antibiotic prescriptions, the numerators of prescription rates were the weighted number of prescriptions that containing one or multiple antibiotics, and the denominator was total weighted number of antibiotic prescriptions. For narrow spectrum and broad-spectrum antibiotics, the numerator was the weighted number of prescriptions that contained each specific antibiotic and the denominator was the weighted number of prescriptions that contained only one antibiotic. For multi-antibiotic prescriptions, the numerator was the weighted number of prescriptions that contained each specific antibiotic combination and the denominator was the weighted number of prescriptions that contained multiple antibiotics.

### Antibiotic prescribing patterns and symptom management medication for adults

Of 5240 prescriptions for acute bronchitis patients aged 18 and above, 46.4% included antibiotics and 65.2% included symptom management drugs (Table 3). Traditional Chinese Medicines for respiratory system were identified as the most commonly prescribed symptom management drug categories (35.2%), followed by expectorants (26.8%) and antihistamines (8.9%). Routine treatment of uncomplicated acute bronchitis with antibiotics was not recommended in China, US, and UK. Penicillins, macrolides, cephalosporins, and fluoroquinolones were recommended as treatment options for certain patients by the Chinese national guideline. Amoxicillin, clarithromycin, and erythromycin were recommended by the UK for certain patients. Cough suppressants were recommended for cough symptom treatment in China, US, and UK, while inhaled or systemic corticosteroids for cough management were not recommended in all the countries (Supplementary Table 2).

**Table 3:** Prescribing of antibiotic and symptom management medications for adult acute bronchitis at primary healthcare facilities in China, by urban–rural location and whether medications were included in clinical guidelines.

Drug subgroup/substance	Prescription rate (weighted, %)				Clinical guideline recommendations^a^		
	Total	Urban	Rural	*p*	CN	US	UK
**Patients treated with antibiotics**	46.4	39.3	55.9	***			
Penicillins, only	11.4	9.1	14.3	***	√	×	
Amoxicillin	7.6	5.5	10.3	***			√
Other	3.8	3.6	4.0				×
Macrolides, only	5.9	3.6	9.0	***	√	×	
Clarithromycin	0.2	0.2	0.2				√
Erythromycin	2.7	1.3	4.6	***			√
Other	3.0	2.1	4.1	***			×
Cephalosporins, only	19.1	19.3	18.9		√	×	×
Fluoroquinolones, only	3.7	2.2	5.7	***	√	×	×
Other antibiotics, only	4.0	4.0	4.0		×	×	×
Multiple antibiotics	2.3	1.0	4.1	***	×	×	×
**Patients treated with symptom management drugs**	**65.2**	**61.7**	**70.1**	***			
Cough suppressants	2.1	1.7	2.6	*	√	√	√
Expectorants	26.8	22.0	33.3	***	√	×	√
Antihistamines	8.9	4.7	14.5	***	√		×
First generation	0.2	0.2	0.2			√	
Second/third generation	8.7	4.5	14.4	***		–	
Combination of the 3 categories above	2.0	0.8	3.7	***	√	–	–
Corticosteroids	5.7	5.4	6.1		×	×	×
Decongestants	0.0	0.0	0.1		–	√	×
Xanthines	7.2	4.6	10.7	***	–	–	×
Anticholinergics	0.2	0.3	0.0	**	–	–	×
Vitamin C	3.7	2.2	5.7	***	–	–	–
Other western respiratory drugs	5.8	4.1	8.0	***	–	–	–
TCM for respiratory system	35.2	35.3	35.0		–	–	–

*CN* China, *US* the United States, *UK* the United Kingdom.

*p < 0.05; **p < 0.01; ***p < 0.001.

^**a**^√, Recommended/recommended under certain circumstances; ×, not recommended/not recommended under certain circumstances; −, not mentioned. Detailed comparison across the national guidelines is summarized in Appendix 2.

Overall, 17.3% of the adult patients in our sample were prescribed only antibiotics for acute bronchitis, while 36.1% were prescribed only symptom management drugs, 29.1% were prescribed both antibiotics and symptom management drugs, and 17.5% were not prescribed with drugs for acute bronchitis (Table 4). The overall rate of compliance with the Chinese national clinical guideline for acute bronchitis management was 31.0%. Community centers showed better compliance than the township hospitals (33.2% vs. 28.0%, *p* = 0.000). The overall compliance rates of Chinese primary healthcare prescribers following clinical guidelines of the US and the UK were 18.5% and 27.4%, respectively.

**Table 4:** Overall prescribing patterns and guideline compliance for adult acute bronchitis at primary healthcare in China.

Drug subgroup/substance	Prescription rate (weighted, %)				Guideline compliance rate, CN (weighted, %)				Guideline compliance rate, US (weighted, %)				Guideline compliance rate, UK (weighted, %)			
	Total	Urban	Rural	*p*	Total	Urban	Rural	*p*	Total	Urban	Rural	*p*	Total	Urban	Rural	*p*
Antibiotics, only	17.3	17.2	17.4		0.8	1.5	0.0	*	0.0	0.0	0.0	–	0.0	0.0	0.0	–
Symptom management drugs, only	36.1	39.5	31.5	***	37.0	29.9	49.1	***	2.7	3.0	2.3		27.5	24.1	33.3	***
Antibiotics+symptom management drugs ^a^	29.1	22.1	38.6	***	0.0	0.0	0.0	–	0.0	0.0	0.0	–	0.0	0.0	0.0	–
Not treated with drugs	17.5	21.2	12.6	***	100.0	100.0	100.0	–	100.0	100.0	100.0	–	100.0	100.0	100.0	–
Total	100.0	100.0	100.0	–	31.0	33.2	28.0	***	18.5	22.3	13.3	***	27.4	30.7	23.1	***

*CN* China, *US* the United States, *UK* the United Kingdom.

*p < 0.05; **p < 0.01; ***p < 0.001.

^**a**^Prescriptions were treated as complied if both antibiotics and symptom management drugs were complied with guidelines.

## Discussion

In light of the paucity of studies investigating detailed outpatient treatment patterns for acute bronchitis in primary healthcare settings in China, we evaluated prescribing patterns of antibiotics and symptom management medications for patients with acute bronchitis treated in 214 primary healthcare settings across China.

Our results indicated that, in 2017, Chinese primary healthcare clinicians prescribed antibiotics for nearly half of outpatients with acute bronchitis, which was a lower rate than those in the studies conducted in Italy (73.5%)^13^, US (74%)^14^, Belgium (69.0%)^15^, Spain (58.6%)^16^, Japan (52.8%)^17^, and Netherlands (51.0%)^18^, and was also lower than previous studies in China (77.5–95.2%)^9,11^. However, our rate was slightly higher than the rate showed in the study conducted in Swiss (41.5%)^19^. One study conducted in England found that 82% of the consultations for acute bronchitis resulted in an antibiotic prescription on the same day, among which only 13% of the patients was necessary for antibiotic treatment according to guidelines and expert elicitations^20^. Another study showed that 85.6% of the antibiotic treatment duration exceeded guideline recommendations in primary care in England^7^. The relatively lower prevalence in our study could be due to the changing patterns of antibiotic use in China^21^, or to our limiting inclusion of acute bronchitis cases without any other infectious diagnosis. The higher rates of antibiotic prescribing in previous studies may be driven by misconceptions of clinicians and patients^22,23^. A Cochrane review suggested that antibiotics were minimally effective for acute bronchitis, with a half-day reduction in cough and no reduction in functional impairment compared to placebo, as well as increasing rates of adverse events^3^. Public perception/misconception of antibiotic efficacy drove inappropriate demand for utilizing antibiotics in the primary outpatient setting in China^24^. Surveys showed that 60% of the population believed that antibiotics were effective for the treatment of acute bronchitis^25^, a belief that varied by educational level^26^. This may partially explain the higher antibiotic prescribing rate in rural and the less developed western region in our study.

Among all single-antibiotic prescriptions, 91.5% were broad-spectrum formulations, and second- and third-generation cephalosporins were the most prescribed categories. This was consistent with previous findings in Chinese hospitals for treating respiratory tract infections^9,10^. This preference for broad-spectrum cephalosporins was also found in the US and European countries^27^. As noted in previous studies, cephalosporins should be avoided when a narrow-spectrum antibiotic would be effective^28^. Inappropriate antibiotic use accelerated the development of antibiotic resistance such as methicillin-resistant staphylococci^29^. Cephalosporin overuse in Chinese primary healthcare outpatient settings may be largely due to the requirement of mandatory skin testing before prescribing either oral or injectable penicillins for all patients^30^. Ideally, skin testing can increase the number of incidents in which penicillin can be safely administered rather than alternative broad-spectrum antibiotics^31^. However, in clinical practice, overestimation of penicillin allergy rates^32,33^, along with the inconvenience and unreliable results of penicillin skin testing, may actually impel some primary healthcare clinicians to replace penicillins with cephalosporins or other broad-spectrum antibiotics^31,33^.

Supportive care and symptom management were the mainstay of treatment for adult patients with acute bronchitis^34^. Our study provided a comprehensive overview of prescribing patterns of symptom management drugs for acute bronchitis patients in Chinese primary healthcare settings; however, evidence supporting these symptomatic therapies was limited according to national clinical guidelines^5,34,35^. For example, inhaled or systemic corticosteroids for cough management were not recommended as there were no data to support their therapeutic effect for acute cough caused by acute bronchitis^34–36^. Insufficient quality data were support to recommend the routine use of Chinese herbs for acute bronchitis and other respiratory conditions according to a Cochrane review^37^.

Barriers to evidence-based treatment of acute bronchitis in Chinese primary healthcare settings include three main issues. First, most primary healthcare clinicians in China lacked of training for evidence-based thinking, and they mainly practiced based on their experience without paying attention to the latest evidence^38^. Second, even if familiar with evidence-based practice, primary healthcare clinicians may have difficulties getting access to updated clinical guidelines, as most of the guidelines were published in academic journals and cannot be obtained for free. Third, Chinese guidelines were sometimes vaguer in expression and more liberal in medication selection than those in other countries (Supplementary Table 2), which made it harder for primary clinicians to understand and to follow the guidelines during daily practice. Furthermore, systems for management and supervision of antibiotic utilization in Chinese primary healthcare settings were generally not in place. Routine national antimicrobial utilization and antimicrobial resistance surveillance networks for large hospitals in China operated for years^39,40^, but similar systems was not established in primary healthcare settings in China^7^.

Our study found that prescribing of antibiotics, especially broad-spectrum ones like cephalosporins, for acute bronchitis remained common in Chinese primary healthcare settings. Antibiotic stewardship and interventions to improve the recognition of inappropriate antibiotic prescribing were urgently needed. Efficient electronic practice advisory tools in prescribing systems^4^, prescriber education^12^, public reporting^11^, shared decision making^41^, delayed antibiotic prescriptions^42^, and involvement of multidisciplinary teams for audit and feedback^12^ were effective measures to improve antibiotic prescribing in previous studies. Primary healthcare clinicians in China may have difficulties in accessing, understanding, and following clinical guidelines in practice. Therefore, tailored training and continuous education for primary healthcare clinicians that targeted at evidence-based treatment based on clinical guidelines could contribute to promoting prescribing quality. In addition, routine national antibiotic stewardship systems based on real-time utilization data would be beneficial in improving antibiotic use in Chinese primary healthcare settings.

Several limitations to this study should be noted. First, we selected acute bronchitis prescriptions from a national survey sample, which was designed to cover the full range of primary care diagnoses. The study sample was not designed to be fully representative of all acute bronchitis patients at primary healthcare settings in China. Second, we adopted overall population counts by using levels of economic status and urban–rural areas as weights to adjust the analyses to better reflect national data. However, the prevalence of acute bronchitis was not equally distributed in all regions of China, and we weighted our estimates based on population. This may lead to over- or under-weighting of the sample estimates. However, given that prescriptions were sampled consistently in all facilities, the prevalence of acute bronchitis cases in the sample should be reasonably representative of their overall prevalence at the time of the study. Third, due to the limitations of the data access, many factors that may affect the quality of prescribing, such as characteristics and knowledge of clinicians, laboratory tests, patient socio-economic characteristics, medical history, drug affordability, cigarette smoking, and environmental pollution were not measured. Fourth, although we excluded the patients who also had a diagnosis of other infections together with acute bronchitis, we cannot assure that the drugs were prescribed only for acute bronchitis.

In conclusion, clinicians prescribed antibiotics for nearly half of outpatients with acute bronchitis in Chinese primary healthcare settings, a level far higher than indicated by guidelines, with particular overuse of broad-spectrum antibiotics. Medications for symptom management were also commonly used in practice. In all, more than two-thirds of the acute bronchitis treatments for adults were not consistent with guideline recommendations. Targeted interventions to improve primary healthcare practice and systems to increase detection of inappropriate antibiotic prescribing were urgently needed for Chinese primary clinicians, especially in western rural areas.

## Methods

### Study design

We conducted a nationwide cross-sectional survey of primary healthcare settings in China to collect outpatient prescriptions from January 2017 to December 2017. Ethics approval was obtained from Peking University Institution Review Board.

### Sampling and data collection

We systematically selected community healthcare centers in urban areas and township hospitals in rural areas across China. First, we classified all 408 cities in China into four levels of economic status according to GDP per capita in 2016. We then selected nine provinces (East region: Beijing, Shandong, Guangdong; Central region: Jilin, Anhui, Jiangxi; West region: Qinghai, Sichuan, Yunnan) out of 31 provinces in mainland China to conduct the survey. Among these provinces, 42 cities were randomly selected according to the four economic strata. Finally, in each selected city, two community healthcare centers and nine township hospitals were selected randomly based on the ratio of these two facility types nationally in 2017 (ref. ^43^).

In each sample facility, 50 outpatient prescriptions were randomly selected from patient encounters that took place on the second Tuesday of each month. Prescription data including visit date, patient demographic characteristics, diagnoses, and medications were digitally extracted from outpatient records and verified by two investigators.

### Inclusion criteria

Patients diagnosed with acute bronchitis were eligible for this study. Patients who also diagnosed with other infections were excluded (Supplementary Table 1).

### Measurements

We classified antibiotics according to Anatomical Therapeutic and Chemical classification J01 (ref. ^44^). Narrow-spectrum antibiotics consisted of narrow-spectrum penicillins, first-generation cephalosporins, nitroimidazoles, tetracyclines, and sulfonamides. Broad-spectrum antibiotics included broad-spectrum penicillins, advanced-generation cephalosporins, macrolides, quinolones, lincosamides, aminoglycosides (except for streptomycin), and other antibiotics (e.g., fosfomycin)^45^.

The primary outcome was APR. The numerator was the weighted number of acute bronchitis prescriptions that contained at least one antibiotic, and the denominator was the total weighted number of acute bronchitis prescriptions. Secondary outcomes included the prescription rate of different antibiotic classes, the proportions of different treatments for acute bronchitis, and the overall guideline compliance rate. We presented the detailed comparisons of national guidelines for treatment strategies of adult acute bronchitis in China, US, and UK in Supplementary Table 2, and the definition of our measure of overall guideline compliance in Supplementary Table 3. Independent variables in our analysis included economic status and region of the city, urban/rural location of the clinic, and patient gender and age.

### Statistical analysis

STATA 14.0 was used for all statistical analysis. We used generalized estimating equations to generate population-weighted comparisons, standardized margins to calculate mean adjusted proportions, and chi square test to compare the urban–rural differences. We weighted sample estimates to reflect the distribution of economic level and urban–rural status in the national population. The level of statistical significance was defined as *p* < 0.05 (two sided).

### Reporting summary

Further information on research design is available in the Nature Research Reporting Summary linked to this article.

## Supplementary information

Supplementary Information

Reporting Summary

## Data Availability

Data of this study are available upon reasonable request from corresponding author. The data are not publicly available due to restrictions, e.g., their containing information that could compromise the privacy of research participants.

## Code availability

Code of this study are available upon reasonable request from corresponding author.
