# Supplementary material for: Outpatient prescribing pattern for acute bronchitis in primary healthcare settings in China
Source: NPJ Prim Care Respir Med. 2021 May 10;31:24. doi: 10.1038/s41533-021-00234-y (PMC8111021; doi:10.1038/s41533-021-00234-y)
Supplement: Supplementary file 1 — Supplementary Information [file 41533_2021_234_MOESM1_ESM.pdf]

Supplementary information

Supplementary Figure 1: Study Flow Diagram

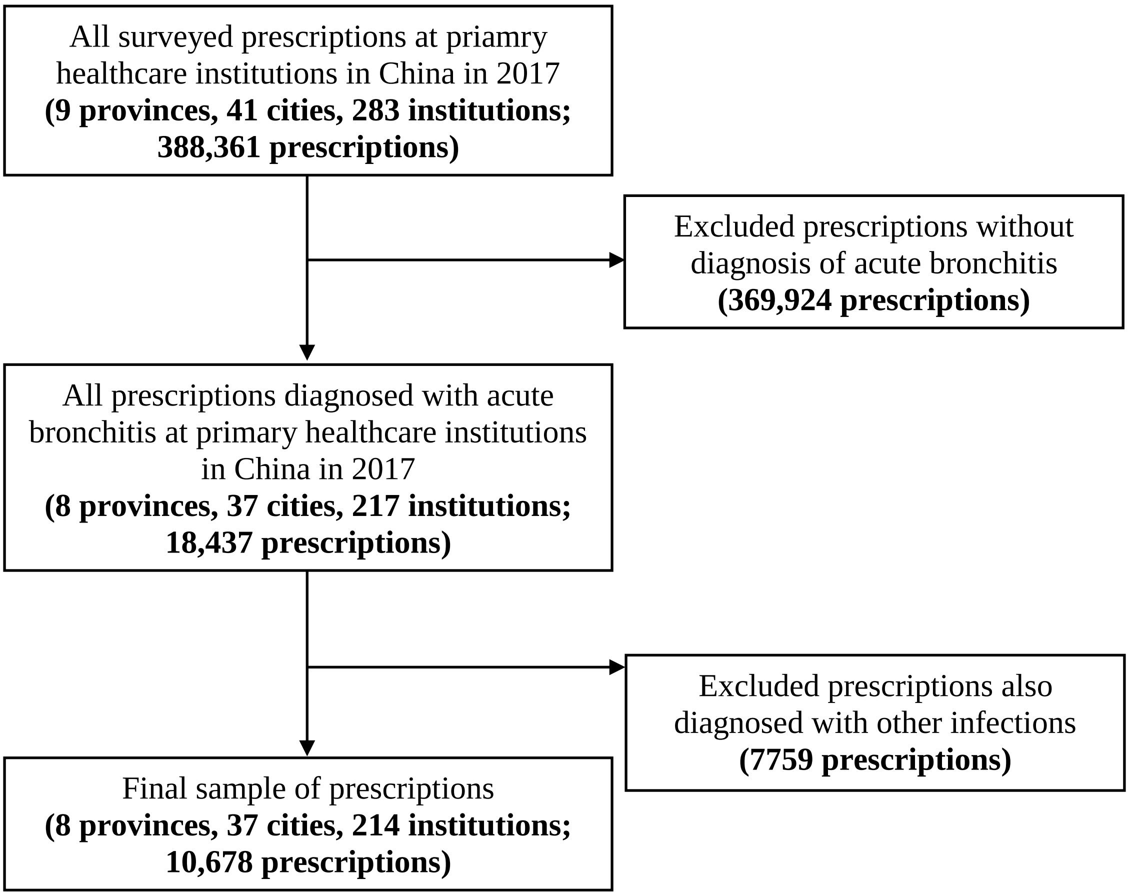

Supplementary Table 1: Inclusion and exclusion of prescriptions by diagnostic category with corresponding International Classification of Diseases, Tenth Revision (ICD-10) diagnosis codes.

**Diagnosis**	**Corresponding ICD-10 Codes**[1]	**Prescription Number**
Inclusion		
1. Acute bronchitis	Acute bronchitis (J20); Bronchitis, not specified as acute or chronic (J40) ***Excluding*** simple and mucopurulent chronic bronchitis (J41); Unspecified chronic bronchitis (J42)	18,437 included
Exclusion		
2. Nervous system infections	Bacterial meningoencephalitis and meningomyelitis, not elsewhere classified (G04.2); Bacterial meningitis, not elsewhere classified (G00)	0 excluded
3. Eye and adnexa infections	Conjunctivitis (H10); Keratitis (H16); Purulent endophthalmitis (H44.0)	408 excluded
4. Ear and mastoid process infections	Otitis externa (H60.0-H60.5); Otitis media (H65-H66)	16 excluded
5. Circulatory system infections	Acute and subacute infective endocarditis (I33)	0 excluded
6. Other respiratory system infections	Acute upper respiratory infections (J00-J06); Influenza and pneumonia (J09-J18); Other diseases of upper respiratory tract (J30-J39); Emphysema (J43); Other chronic obstructive pulmonary disease (J44)	6,986 excluded
7. Digestive system infections	Peritoneal adhesions (K66.0); Diseases of pulp and periapical tissues (K04); Gingivitis and periodontal diseases (K05)	97 excluded
8. Skin and subcutaneous tissue infections	Infections of the skin and subcutaneous tissue (L00-L08)	155 excluded
9. Musculoskeletal system infections	Arthritis (M01.3); Bursitis (M73.0); Osteomyelitis (M90.2*); Synovitis (M68.0); Tenosynovitis (M68.0)	22 excluded
10. Genitourinary system infections	Tubulo-interstitial nephritis (N10-N12); Cystitis (N30); Urinary tract infection, site not specified (N39.0); Inflammatory diseases of female pelvic organs (N70-N77)	32 excluded
11. Sexually transmitted infections	Infections with a predominantly sexual mode of transmission (A50-A64)	1 excluded
12. Other bacterial infections	Other and unspecified gastroenteritis and colitis of infectious origin (A09.0); Other bacterial diseases (A30-A49)	42 excluded
Final sample		10,678

Supplementary Table 2: Pharmaceutical treatment recommendations for adult acute bronchitis according to the guidelines in China, U.S. and U.K.

**Pharmaceutical Treatment**	**China**[2]	**U.S.** [3,4]	**U.K.*** [5]
Antibiotics	Routine treatment of uncomplicated acute bronchitis with antibiotics is not recommended.	Routine treatment of uncomplicated acute bronchitis with antibiotics is not recommended.	No routine antibiotic for acute cough associated with uncomplicated acute bronchitis.
	Antibiotics may be benefit for some specific population after carefully evaluating potential benefit and adverse reaction, including people over 65 with 2 or more of, or over 80 with 1 or more of: hospitalization in previous year, type 1 or 2 diabetes, history of congestive heart failure, current use of oral corticosteroids. Options for antibiotics includes **penicillins, cephalosporins, macrolides or fluoroquinolones**.	When pertussis is suspected as the etiology of cough, initiation of a macrolide antibiotic is recommended as soon as possible to reduce transmission.	For people who are identified as systemically very unwell, offer an immediate antibiotic prescription. For people who are identified as at higher risk of complications, consider an immediate antibiotic prescription or a back-up antibiotic prescription. Higher risk of complications includes people with pre-existing comorbidity; people over 65 with 2 or more of, or over 80 with 1 or more of: hospitalization in previous year, type 1 or 2 diabetes, history of congestive heart failure, current use of oral corticosteroids.<br>- **First choice antibiotic:** doxycycline, 200 mg on first day, then 100 mg once a day for 4 days (5-day course in total).<br>- **Alternative first choices:** amoxicillin 500 mg three times a day/ clarithromycin 250 mg to 500 mg twice a day/ erythromycin 250 mg to 500 mg four times a day or 500 mg to 1,000 mg twice a day for 5 days.
Symptom management	1. Options for symptomatic therapy include:<br>• Cough suppressants (eg. dextromethorphan, pentoxyverine, benproperine)<br>• Expectorants (eg. bromhexine, ambroxol, N-acetylcysteine)<br>• Antispasmodic and Antihistamines (eg. salbutamol, aminophylline, chlorpheniramine)<br>2. Inhaled or systemic corticosteroids for cough management are not recommended.	1. Options for symptomatic therapy include:<br>• Cough suppressants (codeine, dextromethorphan);<br>• First-generation antihistamines (diphenhydramine);<br>• Decongestants (phenylephrine).<br>Evidence supporting specific symptomatic therapies is limited.	1. Some people may wish to try the following self-care treatments, which have limited evidence of some benefit for the relief of cough symptoms:<br>• Pelargonium (a herbal medicine)<br>• Over-the-counter cough medicines containing the expectorant guaifenesin<br>• Over-the-counter cough medicines containing cough suppressants, except codeine, (in people who do not have a

		2. Expectorants and inhaled adrenergics are not recommended for routine use.	persistent cough, such as in asthma, or excessive secretions).<br>Limited evidence suggests that antihistamines, decongestants and codeine-containing cough medicines do not help cough symptoms.<br>2. Oral/inhaled bronchodilators (eg. salbutamol) or oral/inhaled corticosteroids should not be used to treat people with symptoms of an acute cough associated with acute bronchitis unless the person has an underlying airways disease, such as asthma.<br>3. Mucolytic (eg. acetylcysteine or carbocisteine) should not be used to treat people with symptoms of an acute cough associated with acute bronchitis

* This is in the NICE treatment guideline for acute cough.

Supplementary Table 3: Study definition of guideline compliance according to the guidelines in China, U.S. and U.K.

**Compliance with guidelines**	**China**	**U.S.**	**U.K.**
Antibiotics	1. Not prescribed with antibiotics	1. Not prescribed with antibiotics	1. Not prescribed with antibiotics
	2. Prescribed penicillins, cephalosporins, macrolides or fluoroquinolones for people over 65 with 2 or more of, or over 80 with 1 or more of: diagnosed also with type 1 or 2 diabetes, congestive heart failure, current use of oral corticosteroids.		2. Prescribed doxycycline, amoxicillin, clarithromycin, erythromycin for people over 65 with 2 or more of, or over 80 with 1 or more of: diagnosed also with type 1 or 2 diabetes, congestive heart failure, current use of oral corticosteroids.
Symptom management	Prescribed:<br>1. Cough suppressants;<br>2. Expectorants;<br>3. Antihistamines;<br>4. Combination of the 3 categories above.	Prescribed:<br>1. Cough suppressants (codeine, dextromethorphan);<br>2. First-generation antihistamines (diphenhydramine);<br>3. Decongestants (phenylephrine).	Prescribed:<br>1. Over-the-counter cough medicines containing the expectorant guaifenesin;<br>2. Over-the-counter cough medicines containing cough suppressants, except codeine.

Supplementary References

[1] World Health Organization. ICD-10 online version 2019. https://icd.who.int/browse10/2019/en. Accessed on April 2 2020.
[2] Chinese Medical Association. Guidelines for the Diagnosis and Treatment of Acute Bronchitis at the Primary Level (2018). Chin J Pract. 2019;18(4):314-317.
[3] Centers for Disease Control and Prevention. Adult Treatment Recommendations. https://www.cdc.gov/antibiotic-use/community/for-hcp/outpatient-hcp/adult-treatment-rec.html. Accessed April 14, 2020.
[4] Albert RH. Diagnosis and treatment of acute bronchitis. Am Fam Physician. 2010;82(11):1345-50.
[5] National Institute for Health and Care Excellence. Cough (acute): antimicrobial prescribing. https://www.nice.org.uk/guidance/ng120. Accessed April 14, 2020.
