# Supplementary material for: Outpatient prescribing pattern for acute bronchitis in primary healthcare settings in China
Source: NPJ Prim Care Respir Med. 2021 May 10;31:24. doi: 10.1038/s41533-021-00234-y (PMC8111021; doi:10.1038/s41533-021-00234-y)
Supplement: Supplementary file 2 — Reporting Summary [file 41533_2021_234_MOESM2_ESM.pdf]

nature research

Corresponding author(s): NPJPCRM-01172

Last updated by author(s): Mar 22, 2021

Reporting Summary

Nature Research wishes to improve the reproducibility of the work that we publish. This form provides structure for consistency and transparency in reporting. For further information on Nature Research policies, see our Editorial Policies and the Editorial Policy Checklist.

Statistics

For all statistical analyses, confirm that the following items are present in the figure legend, table legend, main text, or Methods section.

n/a	Confirmed	
☒	☐	The exact sample size (*n*) for each experimental group/condition, given as a discrete number and unit of measurement
☒	☐	A statement on whether measurements were taken from distinct samples or whether the same sample was measured repeatedly
☐	☒	The statistical test(s) used AND whether they are one- or two-sided<br>*Only common tests should be described solely by name; describe more complex techniques in the Methods section.*
☒	☐	A description of all covariates tested
☐	☒	A description of any assumptions or corrections, such as tests of normality and adjustment for multiple comparisons
☐	☒	A full description of the statistical parameters including central tendency (e.g. means) or other basic estimates (e.g. regression coefficient) AND variation (e.g. standard deviation) or associated estimates of uncertainty (e.g. confidence intervals)
☐	☒	For null hypothesis testing, the test statistic (e.g. *F*, *t*, *r*) with confidence intervals, effect sizes, degrees of freedom and *P* value noted<br>*Give P values as exact values whenever suitable.*
☒	☐	For Bayesian analysis, information on the choice of priors and Markov chain Monte Carlo settings
☒	☐	For hierarchical and complex designs, identification of the appropriate level for tests and full reporting of outcomes
☒	☐	Estimates of effect sizes (e.g. Cohen's *d*, Pearson's *r*), indicating how they were calculated

Our web collection on statistics for biologists contains articles on many of the points above.

Software and code

Policy information about availability of computer code

Data collection	Code of this study are available upon reasonable request from corresponding author.
Data analysis	Code of this study are available upon reasonable request from corresponding author.

For manuscripts utilizing custom algorithms or software that are central to the research but not yet described in published literature, software must be made available to editors and reviewers. We strongly encourage code deposition in a community repository (e.g. GitHub). See the Nature Research guidelines for submitting code & software for further information.

Data

Policy information about availability of data

All manuscripts must include a data availability statement. This statement should provide the following information, where applicable:

- Accession codes, unique identifiers, or web links for publicly available datasets
- A list of figures that have associated raw data
- A description of any restrictions on data availability

Data of this study are available upon reasonable request from corresponding author. In order to ensure full anonymity, confidentiality and data protection for the participants, the full survey data cannot be made accessible to the public.

Field-specific reporting

Please select the one below that is the best fit for your research. If you are not sure, read the appropriate sections before making your selection.

☐ Life sciences ☒ Behavioural & social sciences ☐ Ecological, evolutionary & environmental sciences

For a reference copy of the document with all sections, see nature.com/documents/nr-reporting-summary-flat.pdf

Behavioural & social sciences study design

All studies must disclose on these points even when the disclosure is negative.

Study description	We conducted a nationwide cross-sectional survey of primary healthcare institutions in China, to collect outpatient prescriptions from January 2017 to December 2017. Ethics approval was obtained from Peking University Institution Review Board.
Research sample	We systematically selected community healthcare centers in urban areas and township hospitals in rural areas across China. First, we classified all 408 cities in China into 4 levels of economic status according to GDP per capita in 2016. We then selected 9 provinces (East region: Beijing, Shandong, Guangdong; Central region: Jilin, Anhui, Jiangxi; West region: Qinghai, Sichuan, Yunnan) of the 31 in mainland China to conduct the survey. Among these provinces, 42 cities were randomly selected according to the 4 economic strata. Finally, in each selected city, two community health centers and 9 township hospitals were selected randomly based on the ratio of these facility types nationally in 2017.
Sampling strategy	In each sample facility, 50 outpatient prescriptions were randomly selected from patient encounters that took place on the second Tuesday of each month.
Data collection	Prescription data including visit date, patient demographic characteristics, diagnoses and medications were digitally extracted from outpatient records and verified by two investigators.
Timing	We tried to reach out to all the sample institutions starting from October 2017, inviting them to participate in our survey. And the data collection was finished in April 2018.
Data exclusions	Patients diagnosed with acute bronchitis were eligible for this study; patients who also had a diagnosis of other infections were excluded (Appendix 1)
Non-participation	*State how many participants dropped out/declined participation and the reason(s) given OR provide response rate OR state that no participants dropped out/declined participation.*
Randomization	*If participants were not allocated into experimental groups, state so OR describe how participants were allocated to groups, and if allocation was not random, describe how covariates were controlled.*

Reporting for specific materials, systems and methods

We require information from authors about some types of materials, experimental systems and methods used in many studies. Here, indicate whether each material, system or method listed is relevant to your study. If you are not sure if a list item applies to your research, read the appropriate section before selecting a response.

Materials & experimental systems

n/a	Involved in the study	
☒	☐	Antibodies
☒	☐	Eukaryotic cell lines
☒	☐	Palaeontology and archaeology
☒	☐	Animals and other organisms
☒	☐	Human research participants
☒	☐	Clinical data
☒	☐	Dual use research of concern

Methods

n/a	Involved in the study	
☒	☐	ChIP-seq
☒	☐	Flow cytometry
☒	☐	MRI-based neuroimaging
